# Socioeconomic inequalities in skilled birth attendance and child stunting in selected low and middle income countries: Wealth quintiles or deciles?

**DOI:** 10.1371/journal.pone.0174823

**Published:** 2017-05-03

**Authors:** Kerry L. M. Wong, María Clara Restrepo-Méndez, Aluísio J. D. Barros, Cesar G. Victora

**Affiliations:** 1 International Center for Equity in Health, Post-Graduate Program in Epidemiology, Federal University of Pelotas, Rua Marechal Deodoro, 1160 3° Piso, Pelotas, Brazil; 2 London School of Hygiene & Tropical Medicine, London, United Kingdom; 3 MRC Integrative Epidemiology Unit, School of Social and Community Medicine, University of Bristol, Oakfield House, Oakfield Grove, Bristol, United Kingdom; Universidade de Sao Paulo, BRAZIL

## Abstract

**Background:**

Wealth quintiles derived from household asset indices are routinely used for measuring socioeconomic inequalities in the health of women and children in low and middle-income countries. We explore whether the use of wealth deciles rather than quintiles may be advantageous.

**Methods:**

We selected 46 countries with available national surveys carried out between 2003 and 2013 and with a sample size of at least 3000 children. The outcomes were prevalence of under-five stunting and delivery by a skilled birth attendant (SBA). Differences and ratios between extreme groups for deciles (D1 and D10) and quintiles (Q1 and Q5) were calculated, as well as two summary measures: the slope index of inequality (SII) and concentration index (CIX).

**Results:**

In virtually all countries, stunting prevalence was highest among the poor, and there were larger differences between D1 and D10 than between Q1 and Q5. SBA coverage showed pro-rich patterns in all countries; in four countries the gap was greater than 80 pct points. With one exception, differences between extreme deciles were larger than between quintiles. Similar patterns emerged when using ratios instead of differences. The two summary measures provide very similar results for quintiles and deciles. Patterns of top or bottom inequality varied with national coverage levels.

**Conclusion:**

Researchers and policymakers should consider breakdowns by wealth deciles, when sample sizes allow. Use of deciles may contribute to advocacy efforts, monitoring inequalities over time, and targeting health interventions. Summary indices of inequalities were unaffected by the use of quintiles or deciles in their calculation.

## Introduction

The measurement of socioeconomic inequalities in health has received growing attention in recent years,[1] and will likely be even more prominent during the Sustainable Development Goals (SDG) era from 2015 to 2030. The third SDG (“ensure healthy lives and promote well-being for all at all ages”) has an intrinsic equity component, and the tenth goal (“reduce inequality within and among countries”), albeit focused on economic inequality, also highlights the importance of reducing disparities (https://sustainabledevelopment.un.org). SDG 17 calls for disaggregated analyses of targets according to socioeconomic status and other equity stratifiers.

The availability of survey data in the fields of reproductive, maternal, newborn and child health (RMNCH) has made it possible to systematically document socioeconomic inequalities across countries and over time.[2] Based on principal component analysis of household variables including ownership of assets and housing characteristics,[3] families can be classified into equal sized groups, usually quintiles, each including approximately 20% of all families.

However, there is no specific reason why one should use quintiles rather than say tertiles, quartiles or deciles. Finer breakdowns (e.g. deciles) are as easy to analyze and may help identify subgroups that are at higher risk of poor health or malnutrition, or that present markedly lower intervention coverage than the rest of the population. Because recent national RMNCH surveys tend to have larger sample sizes than in the past, there is good justification for trying out alternative approaches to the use of quintiles.

Our analyses are focused on two indicators that are consistently and strongly associated with socioeconomic position, namely coverage of delivery by a skilled birth attendant (SBA),[2] and prevalence of stunting in children aged under five years.[4] SBA coverage tends to be directly linked to socioeconomic position, thus showing a pro-rich distribution. In contrast, stunting prevalence is usually higher among the poor.

Our analyses have two objectives. First, we compare results obtained through stratification by wealth quintiles and deciles, and assess the degree to which socioeconomic differences may be underestimated by reliance on quintiles. Second, we assess the extent to which stratification by deciles may contribute to a better understanding of patterns of inequality, such as “top inequality” (when the wealthiest group is considerably different from the rest of the population) and “bottom inequality” (when the poorest group stands out from all others).[5]

Analyses are complemented by supporting information in which we estimate the precision for selected RMNCH indicators stratified either by wealth quintiles or deciles, for a range of survey sample sizes.

## Methods

The data used in the present cross-sectional analyses are drawn from Demographic and Health Surveys (DHS), which provide national probability samples of households from low and middle-income countries (www.dhsprogram.com). For each country, we downloaded the most recent publicly available survey (as of October 2014) carried out between 2003 and 2013. The other two eligibility criteria were that (i) the country was classified by the World Bank as a LMIC during survey period and (ii) the sample size included at least 3000 children aged 0–5 years. On average, these samples would include 300 children per decile, a number that was deemed sufficient for the proposed analyses (standard errors of 3 pct points or less for any given level of prevalence).

The institution that commissioned, funded, or carried out the surveys was responsible for ethical approval, as well as ensuring complete confidentiality of survey respondents. For each country, we downloaded the most recent publicly available survey (as of October 2014) carried out between 2003 and 2013 with a sample size of at least 3,000 children.

Data were obtained through standardized interviews with women aged 15–49 years. The DHS wealth index scores are derived from the ownership of country-specific sets of household assets and dwelling characteristics generated by principal component analysis. Each household is then assigned a continuous asset score and samples can then be divided into categories according to these values.

We stratified the sample of each survey down first in five (quintiles) and then in ten (deciles) categories, each containing approximately 20% and 10%, respectively, of the households in the sample. We referred to the first quintile (Q1) as the poorest and the fifth (Q5) as the wealthiest quintile. Similarly, the first decile (D1) was described as poorest and the tenth (D10) as the wealthiest. By definition, D1 and D2 were contained in Q1, and D9 and D10 in Q5.

We began by assessing within-quintile wealth disparities, particularly in Q1 and Q5, by plotting the smoothed average values of the continuous standardized asset index, by centile. These average values were calculated from the mean asset indices per centile across countries. LOWESS smoothing was used.

Two dependent variables were analyzed–stunting prevalence and SBA coverage. Children aged below five were classified as stunted if their height-for-age Z-score was more than two standard deviations below the median value of the World Health Organization Growth Standards (www.who.int/childgrowth). The second outcome was delivery by a skilled birth attendant, such as doctor, nurse, or midwife to assist with a woman's most recent delivery in the five years before the survey. Alternatives to using a skilled birth attendant included being assisted by a traditional birth attendant, untrained health worker, relative, neighbor, or friend (or unassisted deliveries). There was some variability among countries in what types of health professionals were considered as skilled, in the context of each national health system;[6] our results are consistent with the definitions used in each national DHS report. Both for stunting prevalence and SBA coverage, results were stratified by quintiles and deciles of wealth index.

All analyses took into account sampling weights and clustering. The following measures of inequality were calculated:

- extreme group differences for deciles (D1 minus D10) and quintiles (Q1 minus Q5);
- extreme group ratios for deciles (D1 over D10) and quintiles (Q1 over Q5);
- difference between the two poorest (D1 minus D2) and the two richest deciles (D9 minus D10);
- slope indices of inequality (SII) using deciles and quintiles;[7–10] SII are expressed in percent points and represent the difference in the outcome between the two extremes of the wealth scale, obtained by regressing the outcome on the midpoints of the cumulative frequency distribution of the wealth groups–for example, 0.1, 0.3, 0.5, 0.7 and 0.9 for wealth quintiles, when the five quintiles have exactly the same number of children;
- concentration indices (CIX) using deciles and quintiles; [7–10] the CIX is similar to a Gini index, with individual children being ranked according to socioeconomic position on the x axis, and cumulative SBA coverage (or stunting prevalence) on the y axis.

The two extreme group difference measures are further compared using the Wald test (using the “test” command in Stata after running the two models) The difference measures and the SII reflect absolute inequalities, while the ratio measures and CIX signal relative inequalities.[7–10] Graphical displays include equiplots (http://www.equidade.org/equiplot.php) in which each horizontal line shows the results by quintile or decile for a given country.

For characterizing patterns of inequality, (top, bottom and linear [5]), we examined the relationship between national prevalence/coverage and the difference between the outcome measures in D1 and D2 to signal bottom inequality, that is, marked variability at the bottom of the wealth scale. Likewise, the difference between D9 and D10 by national level was used to describe top inequality, or inequality at the wealthier end of the socioeconomic spectrum. We calculated Spearman correlation coefficients between national coverage and the two patterns of inequality and their corresponding two-tailed P levels.

Data analyses were performed using Stata SE version 13 (StataCorp LP, College Station, TX, USA), taking the sample design into account.

## Results

Data were available for 46 surveys for both stunting and SBA. The surveys included in this analysis, with corresponding country name, survey year, UN region and sample size are listed in S1 Table. Because the wealth index score is evaluated at the household level, and because fertility varies with socioeconomic positon, the percentage of children in each decile varies. In the countries studied, the poorest decile included on average 11.7% of the sample, and the richest decile 6.9%.

For each country, we present the prevalence of stunting and SBA coverage at national level. We also present these estimates for each wealth quintile and decile. Detailed results by quintile and decile are shown in S2 Table and S3 Table, respectively.

Table 1 shows that across all available surveys, the average prevalence of stunting was 33% (95%CI: 30–37) with a range from 8% to 58% (Table 1). Low prevalence countries included Colombia, Dominican Republic and Jordan (<15%) and high prevalence countries were Burundi, Madagascar and Timor-Leste, where one in two children aged under five, on average, was stunted.

**Table 1:** Unweighted mean levels and variability of stunting prevalence and SBA coverage in 46 countries, showing national results and extreme quintiles (Q) and deciles (D).

	Group	Mean	95% confidence interval		Minimum	Maximum
**Under-five stunting prevalence (%)**	**National**	33.3	29.6	37.0	7.6	57.9
	**D1 (poorest)**	42.7	39.0	46.5	14.8	68.9
	**D2**	39.9	35.8	44.0	13.1	70.3
	**D9**	24.3	20.5	28.1	2.6	51.8
	**D10 (richest)**	16.9	14.0	19.8	0.1	40.5
	**Q1 (poorest)**	41.3	37.5	45.2	13.9	69.6
	**Q5 (richest)**	21.0	17.7	24.3	1.8	46.5
**Skilled birth attendance (%)**	**National**	62.9	56.1	69.7	10.8	99.6
	**D1 (poorest)**	40.4	32.4	48.3	1.0	99.4
	**D2**	47.1	38.8	55.4	1.8	99.1
	**D9**	85.1	80.2	90.0	26.9	100.0
	**D10 (richest)**	93.0	90.8	95.2	69.8	100.0
	**Q1 (poorest)**	43.6	35.6	51.7	2.1	99.2
	**Q5 (richest)**	88.7	85.0	92.3	46.3	100.0

**Fig 1:**
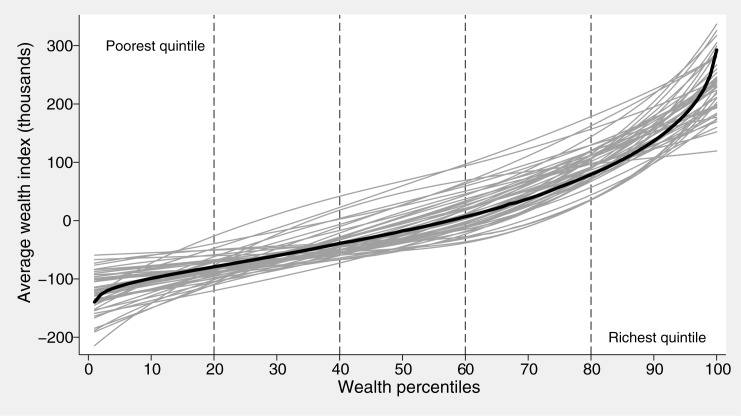
Average values of the standardized asset index, for the 46 countries, according to percentiles.

In Fig 1, we plotted the smoothed average values of the continuous standardized asset index, for the 46 countries by wealth percentile. Within Q5, we can see that the slope of the lines is much steeper than in the other quintiles, for several countries. This implies that there is much more wealth variability within Q5 than in other quintiles. For a few countries, there is also substantial variability in wealth within Q1. Such variability within a given quintile suggests further stratification, e.g. in deciles, may be worthwhile.

**Fig 2:**
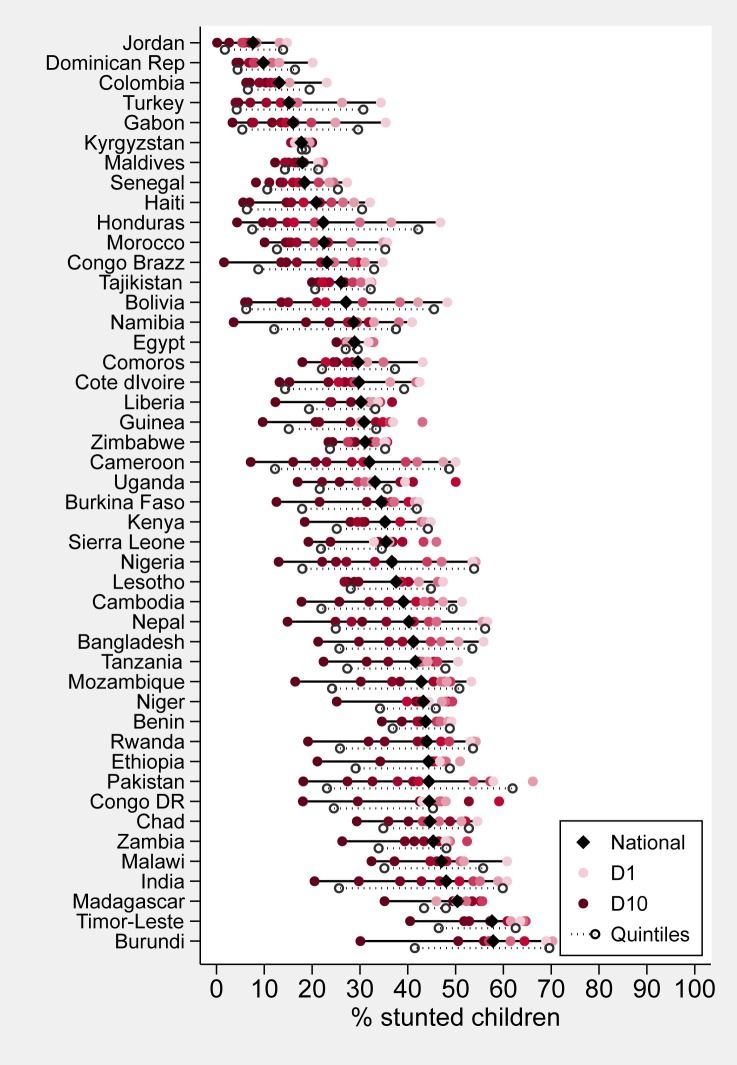
Stunting prevalence by wealth deciles, also showing national levels (black diamonds) and values for the poorest and wealthiest quintiles (hollow circles).

Fig 2 presents equiplots (www.equidade.org/equiplot.php) of stunting prevalence by wealth deciles for all 46 countries. Below each solid line showing the results by decile, national prevalence is shown as a diamond, and prevalence in the poorest (Q1) and richest (Q5) quintiles as hollow circles. Countries are arranged in order of increasing national prevalence. For each country, the widths of the solid line and dashed line represent absolute wealth inequality among wealth deciles and wealth quintiles, respectively. All countries but Kyrgyzstan show higher prevalence among the poor than among the rich. In Egypt and the Maldives, the distance between the extreme deciles is less than 10 percent points; on the other extreme, differences greater than 40 percent points are observed in Honduras, Bolivia, Cameroon, Nigeria, Nepal and India.

Upon visual inspection, distances in stunting prevalence between D1 and D10 tend to be larger than those between Q1 and Q5; the parts of the deciles lines that hang over the quintiles line represent the additional, hidden wealth inequality between D1 and D10 not detected when wealth quintiles were used. This is confirmed by the summary inequality statistics shown in Table 2: only two countries (Senegal and Zimbabwe) have smaller differences between the extreme deciles compared to differences between the extreme quintiles. In Burundi, Madagascar, Mozambique, Namibia and Nepal the decile gap is more than 10 pct points greater than the quintile gap. In 37 of the 46 countries, there is statistical evidence that the extreme-decile differences are wider than the corresponding extreme-quintile differences (p-value < 0.05). On the other hand, in countries such as Pakistan, Sierra Leone and Tajikistan, no statistical evidence of such a difference is found. The same patterns are apparent when comparing the D1/D10 and Q1/Q5 ratios. A particularly high D1/D10 ratio, equal to 106, is observed in Jordan where stunting prevalence in D10 was only 0.1% (Table 2 and S2 Table).

**Table 2:** Summary inequality measures for stunting prevalence, using wealth deciles (D) and quintiles (Q).

Country	Year	National prevalence (%)	Differences between extreme deciles and quintiles (% pts)		p-value (D1-D10 = Q1-Q5)		Ratios between extreme quintiles and deciles		Differences within the poorest and richest quintile (% pts)		Slope index of inequality		Concentration index	
			D1-D10	Q1-Q5			D1/D10	Q1/Q5	D1-D2	D9-D10	D	Q	D	Q
Bangladesh	2011	41.2	34.6	27.8	<0.001	*	2.6	2.1	5.2	8.5	-0.323	-0.323	-0.134	-0.130
Benin	2011	43.7	14.6	11.9	0.271		1.4	1.3	0.7	4.2	-0.140	-0.138	-0.054	-0.051
Bolivia	2008	27.1	41.7	39.3	0.131		7.3	7.3	6.1	-0.6	-0.474	-0.475	-0.328	-0.322
Burkina Faso	2010	34.5	29.8	24.0	0.013	*	3.4	2.3	0.9	9.0	-0.227	-0.223	-0.134	-0.123
Burundi	2010	57.9	38.8	28.1	<0.001	*	2.3	1.7	-1.3	20.5	-0.286	-0.278	-0.091	-0.082
Cambodia	2010	39.1	33.5	27.4	0.026	*	2.9	2.3	4.0	7.9	-0.313	-0.310	-0.145	-0.138
Cameroon	2011	32.0	42.8	36.4	0.012	*	7.0	4.0	2.6	8.9	-0.438	-0.441	-0.251	-0.239
Chad	2004	44.6	25.2	17.9	0.018	*	1.9	1.5	3.4	10.8	-0.201	-0.183	-0.078	-0.069
Colombia	2010	13.1	16.1	12.9	0.017	*	3.3	3.0	7.8	-0.8	-0.148	-0.146	-0.195	-0.193
Comoros	2012	29.6	25.2	15.3	0.025	*	2.4	1.7	11.6	7.1	-0.197	-0.189	-0.110	-0.101
Congo Brazz	2011	23.1	33.2	24.2	<0.001	*	22.1	3.8	3.7	13.0	-0.287	-0.287	-0.236	-0.219
Congo DR	2007	44.5	24.9	20.7	0.340		2.4	1.8	-5.0	11.5	-0.164	-0.155	-0.085	-0.075
Cote dIvoire	2011	29.8	29.3	24.9	0.169		3.2	2.7	6.1	2.1	-0.288	-0.283	-0.180	-0.170
Dominican Rep	2007	9.8	15.4	12.0	0.030	*	4.3	3.7	7.0	-0.5	-0.143	-0.138	-0.244	-0.230
Egypt	2008	28.9	6.7	2.5	0.029	*	1.3	1.1	4.5	3.4	-0.038	-0.028	-0.024	-0.016
Ethiopia	2011	44.3	25.6	19.7	0.012	*	2.2	1.7	-4.2	13.1	-0.172	-0.176	-0.085	-0.077
Gabon	2012	16.0	32.0	24.2	0.001	*	10.6	5.5	10.5	4.0	-0.278	-0.279	-0.298	-0.280
Guinea	2012	30.9	27.2	18.3	<0.001	*	3.8	2.2	6.8	11.0	-0.229	-0.218	-0.151	-0.139
Haiti	2012	20.9	26.5	24.1	0.249		5.7	4.8	3.4	1.3	-0.271	-0.277	-0.244	-0.240
Honduras	2011	22.3	42.5	34.7	<0.001	*	10.9	5.6	10.3	5.4	-0.442	-0.438	-0.342	-0.326
India	2005	48.0	40.3	34.2	<0.001	*	3.0	2.3	1.9	9.3	-0.369	-0.370	-0.150	-0.143
Jordan	2012	7.6	14.6	12.2	0.268		105.9	7.9	1.6	2.5	-0.123	-0.123	-0.300	-0.277
Kenya	2008	35.3	26.3	19.1	0.018	*	2.4	1.8	1.2	12.5	-0.252	-0.243	-0.123	-0.112
Kyrgyzstan	2012	17.8	-3.6	-0.8	0.354		0.8	1.0	-2.9	-2.7	-0.007	-0.005	-0.003	-0.002
Lesotho	2009	37.6	20.0	16.8	0.454		1.7	1.6	5.0	1.4	-0.231	-0.231	-0.107	-0.105
Liberia	2007	30.2	21.5	13.9	0.057		2.7	1.7	1.3	11.7	-0.138	-0.144	-0.103	-0.095
Madagascar	2008	50.4	15.0	4.6	<0.001	*	1.4	1.1	4.2	14.3	-0.027	-0.024	-0.024	-0.017
Malawi	2010	47.1	28.4	20.7	0.028	*	1.9	1.6	9.0	4.8	-0.221	-0.220	-0.083	-0.077
Maldives	2009	18.0	9.0	7.0	0.503		1.7	1.5	-0.1	4.8	-0.105	-0.102	-0.092	-0.090
Morocco	2003	22.5	25.6	22.7	0.158		3.5	2.8	0.9	5.1	-0.286	-0.283	-0.214	-0.204
Mozambique	2011	42.8	36.8	26.6	<0.001	*	3.2	2.1	5.1	13.7	-0.281	-0.282	-0.131	-0.123
Namibia	2006	28.7	37.3	25.5	<0.001	*	11.4	3.1	8.0	15.1	-0.288	-0.280	-0.186	-0.171
Nepal	2011	40.3	41.8	31.2	0.006	*	3.8	2.3	1.0	15.6	-0.395	-0.388	-0.184	-0.163
Niger	2012	43.3	19.2	11.7	0.018	*	1.8	1.3	-2.7	16.6	-0.125	-0.121	-0.056	-0.049
Nigeria	2013	36.7	40.5	35.9	<0.001	*	4.1	3.0	-0.7	9.1	-0.442	-0.445	-0.212	-0.205
Pakistan	2012	44.4	39.7	38.8	0.848		3.2	2.7	-8.3	9.2	-0.443	-0.449	-0.181	-0.175
Rwanda	2010	44.0	34.0	27.8	0.015	*	2.8	2.1	-1.1	12.7	-0.320	-0.316	-0.135	-0.127
Senegal	2012	18.4	13.9	14.8	0.716		2.0	2.4	3.8	-5.1	-0.200	-0.202	-0.183	-0.181
Sierra Leone	2008	35.5	13.7	12.7	0.806		1.7	1.6	-3.2	4.6	-0.125	-0.132	-0.082	-0.080
Tajikistan	2012	26.1	12.1	11.6	0.806		1.6	1.6	-0.3	1.3	-0.136	-0.136	-0.088	-0.086
Tanzania	2010	41.6	28.1	20.5	0.001	*	2.3	1.8	6.3	9.0	-0.210	-0.206	-0.097	-0.091
Timor-Leste	2009	57.6	23.0	16.1	0.005	*	1.6	1.3	2.0	11.3	-0.206	-0.205	-0.061	-0.058
Turkey	2003	15.2	29.8	26.5	0.079		7.5	7.3	8.1	-0.6	-0.351	-0.352	-0.378	-0.370
Uganda	2011	33.2	22.5	14.1	0.055		2.3	1.7	7.0	8.8	-0.140	-0.119	-0.084	-0.069
Zambia	2007	45.3	21.6	14.2	0.028	*	1.8	1.4	-0.5	15.1	-0.157	-0.158	-0.072	-0.068
Zimbabwe	2010	31.1	10.9	11.5	0.682		1.4	1.5	-0.3	-0.8	-0.113	-0.111	-0.067	-0.066

* Significant at 5% level.

In 32 of 46 countries, differences in stunting prevalence at the poor end of the socioeconomic scale (D1-D2) tended to be smaller than differences at the wealthy end (D9-D10). The opposite was observed in the remaining 14. In Burundi, for example, the prevalence of 42% in Q5 hides an important difference between D9 and D10, where prevalences are equal to 51% and 30%, respectively (S2 Table).

Finally, the two summary measures that take the whole socioeconomic distribution into account–the SII and CIX–provide very similar results both for quintiles and deciles, although CIX tends to be very slightly larger when deciles are used.

Turning now to SBA coverage, the average value was 63% (95%CI: 56–70) in all 46 surveys (Table 1). Country-specific national coverage ranged from below 30% in Timor-Leste, Niger, Bangladesh and Chad to almost universal coverage in the Dominican Republic, Jordan and Kyrgyzstan (S3 Table).

**Fig 3:**
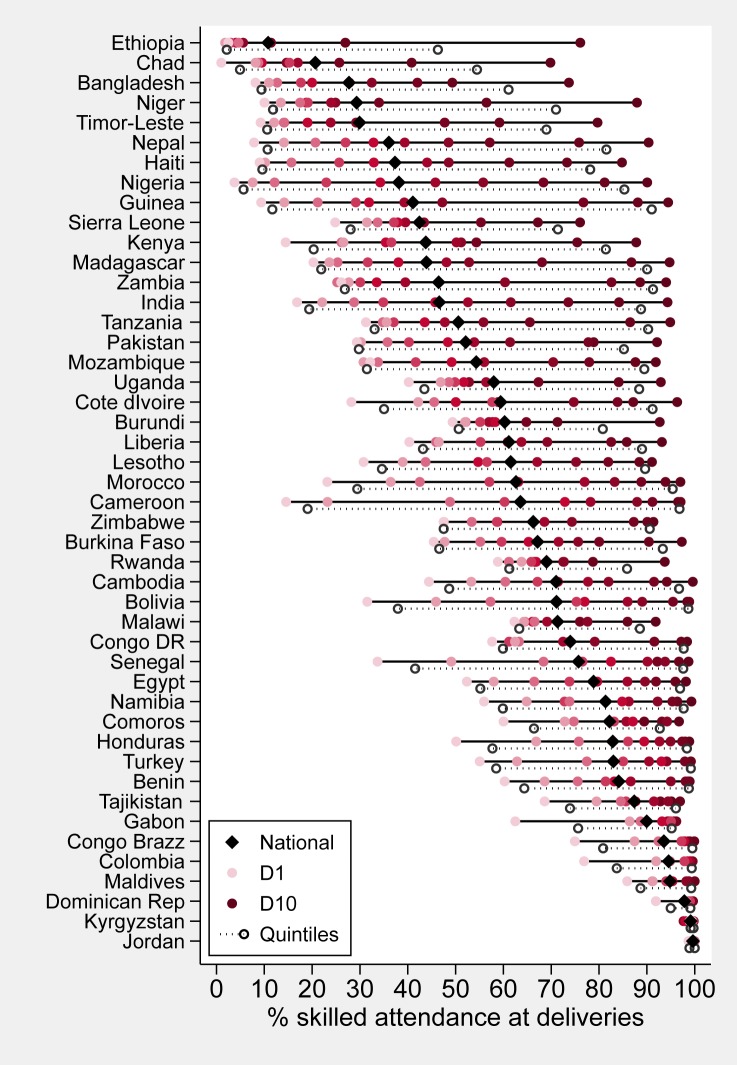
Skilled birth attendance coverage by wealth deciles, also showing national levels (black diamonds) and values for the poorest and wealthiest quintiles (transparent circles).

In contrast with stunting, SBA coverage shows pro-rich patterns in all countries (Fig 3, Table 3 and S3 Table). Except for Kyrgyzstan, Jordan and the Dominican Republic where SBA coverage is virtually universal, all other countries show differences of 10 percent points or greater between the extreme quintiles. The differences between the extreme deciles are even greater, reaching more than 80 percent points in Cameroon, Haiti, Nepal and Nigeria. Except for Kyrgyzstan where national coverage is 99.1%, all other countries appear to have wider gaps between D1 and D10 than between Q1 and Q5. In 28 countries, there is statistical evidence that the two measures are different (p-value of comparison <0.05). The extreme example is Ethiopia, where these gaps are, respectively, 74 and 44 percent points; this discrepancy is largely because the coverage of 46% in Q5 is an average of the widely different coverages of 27% in D9 and 76% in D10. Similar patterns are observed when comparing the D1/D10 ratios, which tend to be more extreme in all countries than the Q1/Q5 ratios, except again for Kyrgyzstan (Table 3).

**Table 3:** Summary inequality measures for skilled birth attendant, using wealth deciles (D) and quintiles (Q).

Country	Year	National coverage (%)	Differences between extreme deciles and quintiles (% pts)		p-value (D1-D10 = Q1-Q5)		Ratios between extreme quintiles and deciles		Differences within the poorest and richest quintile (% pts)		Slope index of inequality		Concentration index	
			D1-D10	Q1-Q5			D1/D10	Q1/Q5	D1-D2	D9-D10	D	Q	D	Q
Bangladesh	2011	27.7	-65.5	-51.7	<0.001	*	0.1	0.2	-2.8	-24.4	0.596	0.586	0.359	0.343
Benin	2011	84.1	-38.7	-34.4	0.009	*	0.6	0.7	-8.4	-0.3	0.461	0.463	0.085	0.082
Bolivia	2008	71.1	-66.8	-60.6	<0.001	*	0.3	0.4	-14.4	0.4	0.735	0.723	0.161	0.157
Burkina Faso	2010	67.1	-51.9	-46.7	<0.001	*	0.5	0.5	-2.3	-6.9	0.518	0.520	0.137	0.133
Burundi	2010	60.3	-43.3	-30.2	<0.001	*	0.5	0.6	-2.6	-21.3	0.327	0.320	0.097	0.089
Cambodia	2010	71.0	-55.2	-48.0	<0.001	*	0.4	0.5	-8.9	-5.4	0.592	0.588	0.133	0.129
Cameroon	2011	63.6	-82.0	-77.8	0.004	*	0.2	0.2	-8.6	0.5	0.855	0.856	0.232	0.227
Chad	2004	20.7	-68.8	-49.5	<0.001	*	0.0	0.1	-7.2	-29.0	0.539	0.517	0.445	0.406
Colombia	2010	94.6	-22.7	-15.7	<0.001	*	0.8	0.8	-15.1	-0.4	0.279	0.265	0.030	0.029
Comoros	2012	82.2	-36.7	-26.3	0.003	*	0.6	0.7	-12.8	-7.3	0.371	0.367	0.068	0.065
Congo Brazz	2011	93.6	-24.1	-18.6	<0.001	*	0.8	0.8	-12.5	0.9	0.277	0.273	0.036	0.034
Congo DR	2007	74.0	-40.8	-37.8	0.098		0.6	0.6	-4.8	-1.2	0.458	0.466	0.106	0.104
Cote dIvoire	2011	59.4	-68.1	-56.1	<0.001	*	0.3	0.4	-14.1	-9.2	0.643	0.629	0.189	0.179
Dominican Rep	2007	97.8	-6.8	-4.1	<0.001	*	0.9	1.0	-5.9	0.7	0.061	0.056	0.008	0.007
Egypt	2008	78.9	-45.8	-41.8	0.002	*	0.5	0.6	-5.6	-2.2	0.519	0.520	0.109	0.105
Ethiopia	2011	10.8	-73.6	-44.2	<0.001	*	0.0	0.0	0.7	-49.0	0.466	0.453	0.628	0.574
Gabon	2012	90.0	-33.2	-19.6	<0.001	*	0.7	0.8	-24.0	-0.8	0.253	0.236	0.044	0.038
Guinea	2012	41.1	-85.1	-79.3	0.001	*	0.1	0.1	-4.8	-6.3	0.811	0.802	0.359	0.347
Haiti	2012	37.3	-75.8	-68.5	0.002	*	0.1	0.1	-1.2	-11.5	0.741	0.736	0.353	0.340
Honduras	2011	82.9	-48.8	-40.7	<0.001	*	0.5	0.6	-16.8	-0.9	0.555	0.548	0.093	0.091
India	2005	46.6	-77.5	-69.4	<0.001	*	0.2	0.2	-5.3	-10.1	0.748	0.746	0.280	0.272
Jordan	2012	99.6	-1.3	-1.1	0.644		1.0	1.0	-0.4	0.0	0.010	0.011	0.001	0.001
Kenya	2008	43.8	-73.3	-61.1	<0.001	*	0.2	0.2	-12.1	-12.4	0.680	0.666	0.262	0.253
Kyrgyzstan	2012	99.1	-0.3	-0.6	0.600		1.0	1.0	0.5	0.1	0.002	0.000	0.000	0.000
Lesotho	2009	61.5	-60.4	-55.1	0.024	*	0.3	0.4	-8.2	-2.7	0.658	0.650	0.183	0.176
Liberia	2013	46.3	-52.8	-45.8	0.008	*	0.4	0.5	-6.2	-7.3	0.534	0.529	0.152	0.146
Madagascar	2008	43.9	-74.5	-68.1	<0.001	*	0.2	0.2	-3.3	-8.1	0.703	0.700	0.286	0.276
Malawi	2010	71.3	-29.5	-25.2	0.008	*	0.7	0.7	-2.0	-5.9	0.279	0.284	0.070	0.068
Maldives	2009	94.8	-12.8	-10.6	0.137		0.9	0.9	-5.3	1.3	0.149	0.150	0.024	0.023
Morocco	2003	62.6	-73.8	-66.0	<0.001	*	0.2	0.3	-13.1	-3.1	0.776	0.767	0.210	0.204
Mozambique	2011	54.3	-59.7	-58.0	0.333		0.4	0.4	1.4	-4.3	0.671	0.675	0.219	0.214
Namibia	2006	81.4	-43.3	-37.9	0.002	*	0.6	0.6	-8.7	-2.9	0.483	0.490	0.095	0.094
Nepal	2011	36.0	-82.5	-70.9	<0.001	*	0.1	0.1	-6.3	-14.5	0.726	0.721	0.345	0.334
Niger	2012	29.3	-77.9	-59.2	<0.001	*	0.1	0.2	-3.4	-31.5	0.594	0.583	0.362	0.341
Nigeria	2013	38.1	-86.4	-79.7	<0.001	*	0.0	0.1	-3.8	-8.9	0.863	0.858	0.399	0.388
Pakistan	2012	52.1	-62.8	-55.4	0.005	*	0.3	0.3	-0.9	-13.2	0.640	0.638	0.216	0.207
Rwanda	2010	62.2	-34.9	-24.6	<0.001	*	0.6	0.7	-5.0	-15.0	0.277	0.273	0.071	0.067
Senegal	2012	69.0	-65.0	-56.1	<0.001	*	0.3	0.4	-15.4	-2.0	0.686	0.684	0.140	0.136
Sierra Leone	2008	42.4	-51.2	-43.3	0.012	*	0.3	0.4	-6.6	-8.8	0.447	0.442	0.188	0.180
Tajikistan	2012	87.4	-26.7	-22.1	0.090		0.7	0.8	-10.8	1.4	0.272	0.273	0.049	0.048
Tanzania	2010	50.6	-63.7	-57.2	0.001	*	0.3	0.4	-4.4	-8.4	0.601	0.601	0.215	0.210
Timor-Leste	2009	29.9	-70.7	-58.5	<0.001	*	0.1	0.2	-2.8	-20.6	0.662	0.653	0.383	0.367
Turkey	2003	83.0	-44.1	-40.7	0.081		0.6	0.6	-7.8	0.1	0.549	0.558	0.091	0.091
Uganda	2011	58.0	-52.7	-44.9	<0.001	*	0.4	0.5	-6.7	-8.8	0.478	0.472	0.143	0.136
Zambia	2007	46.5	-67.9	-64.4	0.073		0.3	0.3	-1.6	-5.4	0.711	0.709	0.280	0.272
Zimbabwe	2010	66.2	-43.9	-43.1	0.726		0.5	0.5	-0.1	-1.2	0.527	0.525	0.136	0.131

* Significant at 5% level.

In contrast with stunting, coverage differences at the top of the wealth scale predominated in low-coverage countries, whereas in high-coverage countries the largest differences were at the bottom of the scale (Fig 3).

**Fig 4:**
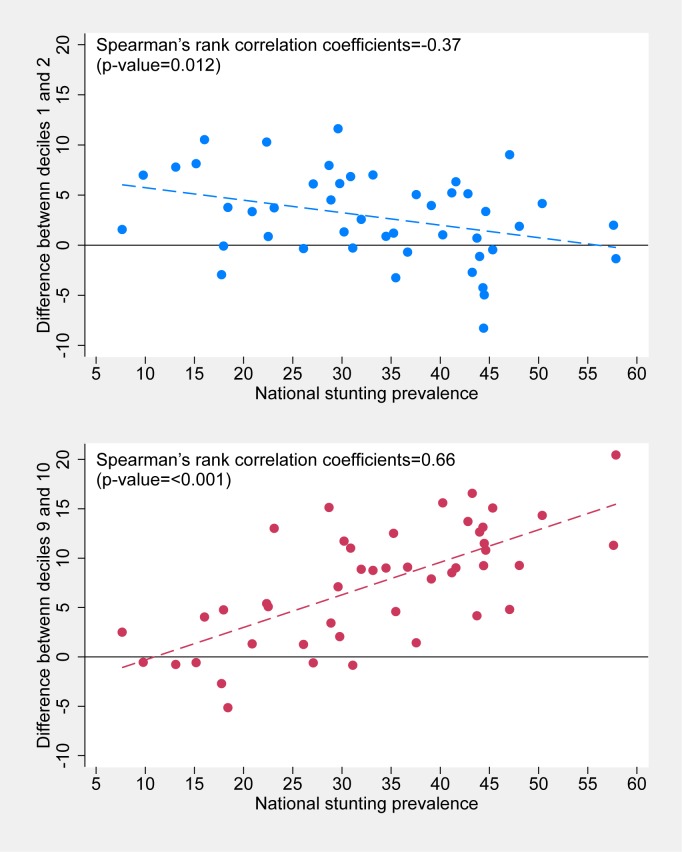
Association between national stunting prevalence and differences between D1 and D2 (top) and between D9 and D10 (bottom).

**Fig 5:**
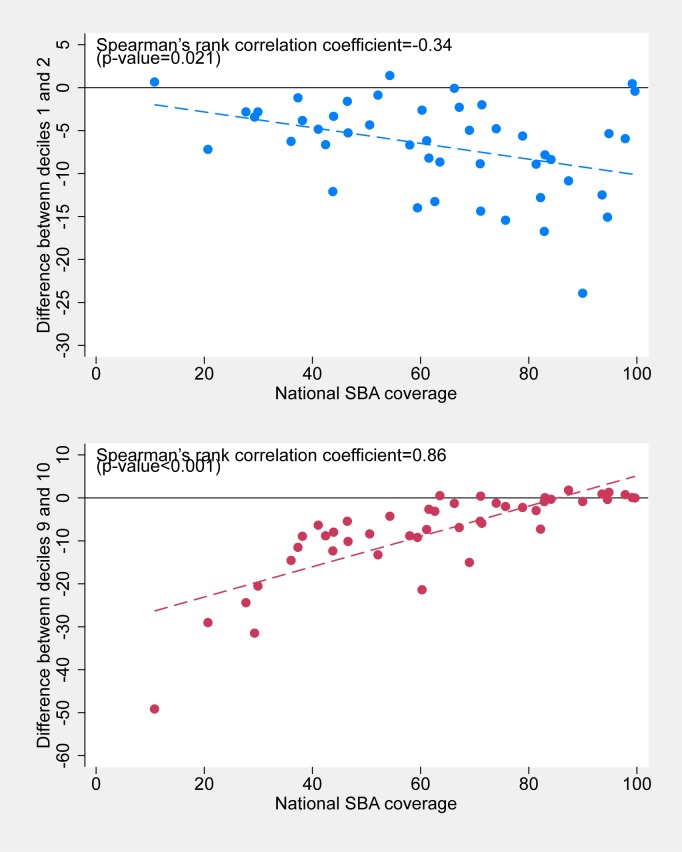
Association between national SBA coverage and differences between D1 and D2 (top) and between D9 and D10 (bottom).

Figs 4 and 5 show the associations between national levels and the degrees of bottom inequality (indicated by differences between D1 and D2 levels) and top inequality (differences between D9 and D10 levels). For stunting (Fig 4), low prevalence countries tend to show bottom inequality, and high prevalence countries show top inequality. In other words, when national prevalence is low, the very poor tend to stand out from the rest with much higher prevalence. Conversely, when national prevalence is high, the very wealthy stand out because of their lower prevalence. The Spearman correlation coefficient between national coverage and bottom inequality was equal to -0.35 (p = 0.02) whereas that with top inequality was 0.69 (p<0.001).

The situation for the patterns of SBA coverage are in opposite direction (Fig 5). When national coverage is low, the rich tend to be way above every other group; and when prevalence increases, the rich tend to lag behind. The Spearman correlation coefficient between national coverage and bottom inequality was equal to -0.38 (p = 0.01) whereas that with top inequality was 0.79 (p<0.001).

## Discussion

We used data from 46 national surveys to assess how using wealth deciles instead of quintiles might affect the interpretation of the magnitudes in inequalities in maternal and child health in low and middle-income countries. We selected two indicators, stunting prevalence among children under the age of five years, and coverage with skilled birth attendants because of their well-established associations with wealth.[2, 4] In virtually all countries, wealth was inversely associated with stunting prevalence, and directly associated with SBA coverage. For both indicators, the associations with wealth deciles tended to be monotonic, that is, prevalence declined and coverage increased for each subsequent decile.

As seen in Fig 1, evidence of higher wealth heterogeneity was observed mostly within the two extreme quintiles, and thus we focused the analyses on these quintiles. We found some advantages to using deciles when sample sizes allow. As might have been predicted from the stepwise association with wealth, comparison of outcomes in the poorest and richest deciles usually resulted in larger differences and ratios than similar comparisons of the extreme quintiles. In some countries, there were important within-quintile differences; for example, in one quarter of the countries studied the difference in SBA coverage was greater than 10 percent points between the poorest and second poorest deciles. Analyses using deciles may be instrumental for advocacy and benchmarking purposes, e.g., reporting inequalities in terms of distance from the best-performing subgroup of the population, as well as for targeting.

Summary indices of inequalities that take into account the whole wealth distribution of the sample rather than only the extreme groups–such as the slope and concentration indices–were not affected by the use of quintiles or deciles in their calculation. This finding is reassuring for scientific audiences who are the main users of such indices, but may be less relevant for policy makers to tend to rely on the more palpable statistics resulting from extreme group comparisons.

Last, deciles were useful for revealing "top" and "bottom" inequalities.[5, 11] When national stunting prevalence was low, bottom inequalities predominated—that is, there tended to be marked gaps between the two poorest deciles. When national prevalence was high, top inequalities tended to prevail, with larger gaps between the wealthiest deciles. Patterns for SBA coverage were in the opposite direction: top inequalities when national coverage was low, yet the rich managed to achieve relatively high coverage levels; and bottom inequalities when national coverage was high but the very poor were yet to be reached. Use of deciles made these patterns more evident than had the analyses been limited to quintiles.

Other than top and bottom inequalities, the use of deciles allows to document patterns of inequality such as universal coverage and a linear pattern. The first refers to a situation in which all subgroups report near 100% coverage. The use of quintiles may show a coverage of say 95% in the poorest group, whereas the use of deciles may reveal that the poorest 10% are the ones responsible for lower coverage in the poorest quintile. In contrast, if coverage is indeed universal, it is obviously irrelevant whether quintiles or deciles are used. The linear pattern (also known as incremental or queueing pattern (10)) describes a steady gradient, moving from the poorest to the richest in approximately equal-sized distances between the groups. When such a pattern is present, using deciles when sample sizes are sufficient may reveal greater disparities than when using quintiles.

Our analyses have limitations. Information on the wealth index, under-five stunting and SBA coverage were not available for all countries. According to the World Bank Income Classification in 2010, our analyses covered 70% (28/40) of low income, 27% (15/56) of lower-middle and 11% (5/45) of upper-middle income counties. We selected the year cut-off of 2010 as this is the median (and mean) survey year of our all included countries. Global results should be interpreted with this limitation in mind. Nevertheless, with 46 different countries this is the largest set of analyses on this topic so far. The use of asset indices to assess socioeconomic position is affected by the choice of assets and poor comparability between urban and rural areas,[12, 13], but such indices are easy to compute and compare well to more complex indicators of wealth.[3, 14, 15] The usefulness such indices is confirmed by our present results showing their strong and usually monotonic, associations with nutrition and coverage. Another limitation is that due to higher fertility among the poor, the actual numbers of children tend to be somewhat larger in the poorer deciles and quintiles than in the rest of the sample.

Finally, use of deciles is limited by the available survey sample size. S1 Supporting Information includes a spreadsheet with sample size calculations for precision according to quintiles and deciles, for different sample sizes.

Our results suggest that, while wealth quintiles are useful for documenting health inequalities, researchers and policymakers should also consider finer breakdowns, such as deciles, when sample sizes are sufficient for such levels of disaggregation. Use of deciles may contribute to advocacy and benchmarking efforts, monitoring inequalities over time, and targeting health interventions.

## Supporting information

S1 Table List of countries, surveys and sample sizes included in the analyses. (DOCX)

S2 Table Prevalence of stunting among children under five years of age, by wealth quintiles and deciles. (DOCX)

S3 Table Coverage with skilled birth attendants, by wealth quintiles and deciles. (DOCX)

S1 Supporting Information Sample size calculations. (DOCX)
