# Supplementary material for: Socioeconomic inequalities in skilled birth attendance and child stunting in selected low and middle income countries: Wealth quintiles or deciles?
Source: PLoS One. 2017 May 3;12(5):e0174823. doi: 10.1371/journal.pone.0174823 (PMC5414946; doi:10.1371/journal.pone.0174823)
Supplement: S1 Table — (DOCX) [file pone.0174823.s001.docx]

**S1 Table. List of countries, surveys and sample sizes included in the analyses.**

|  | **Country** | | **Year** | **World Bank Income Classification in 2010^+^** | **N** |
| --- | --- | --- | --- | --- | --- |
|  | | **Eastern & Southern Africa** | | | |
| 1 | Burundi | | 2010 | Low income | 7742 |
| 2 | Comoros | | 2012 | Low income | 3149 |
| 3 | Ethiopia | | 2011 | Low income | 11654 |
| 4 | Kenya | | 2008 | Low income | 6079 |
| 5 | Lesotho | | 2009 | Lower-middle income | 3999 |
| 6 | Madagascar | | 2008 | Low income | 12448 |
| 7 | Malawi | | 2010 | Low income | 19967 |
| 8 | Mozambique | | 2011 | Low income | 11102 |
| 9 | Namibia | | 2006 | Upper-middle income | 5168 |
| 10 | Rwanda | | 2010 | Low income | 9002 |
| 11 | Tanzania | | 2010 | Low income | 8023 |
| 12 | Uganda | | 2011 | Low income | 7878 |
| 13 | Zambia | | 2007 | Low income | 6401 |
| 14 | Zimbabwe | | 2010 | Low income | 5563 |
|  | | **West & Central Africa** | | | |
| 15 | Benin | | 2011 | Low income | 13407 |
| 16 | Burkina Faso | | 2010 | Low income | 15044 |
| 17 | Cameroon | | 2011 | Lower-middle income | 11732 |
| 18 | Chad | | 2004 | Low income | 5635 |
| 19 | Congo Brazz | | 2011 | Lower-middle income | 9329 |
| 20 | Congo DR | | 2007 | Low income | 8992 |
| 21 | Cote dIvoire | | 2011 | Lower-middle income | 7776 |
| 22 | Gabon | | 2012 | Upper-middle income | 6067 |
| 23 | Guinea | | 2012 | Low income | 7039 |
| 24 | Liberia | | 2013 | Low income | 7606 |
| 25 | Niger | | 2012 | Low income | 12558 |
| 26 | Nigeria | | 2013 | Lower-middle income | 31482 |
| 27 | Senegal | | 2012 | Lower-middle income | 6862 |
| 28 | Sierra Leone | | 2008 | Low income | 5631 |
|  | | **CEE & CIS** | | | |
| 29 | Kyrgyzstan | | 2012 | Low income | 4363 |
| 30 | Tajikistan | | 2012 | Low income | 5013 |
| 31 | Turkey | | 2003 | Upper-middle income | 4533 |
|  | | **East Asia & Pacific** | | | |
| 32 | Cambodia | | 2010 | Low income | 8232 |
| 33 | Timor-Leste | | 2009 | Lower-middle income | 9806 |
|  | | **LAC** | | | |
| 34 | Bolivia | | 2008 | Lower-middle income | 8605 |
| 35 | Colombia | | 2010 | Upper-middle income | 17756 |
| 36 | Dominican Rep | | 2007 | Upper-middle income | 11149 |
| 37 | Haiti | | 2012 | Low income | 7247 |
| 38 | Honduras | | 2011 | Lower-middle income | 10888 |
|  | | **Middle East & North Africa** | | | |
| 39 | Egypt | | 2008 | Lower-middle income | 10872 |
| 40 | Jordan | | 2012 | Lower-middle income | 10360 |
| 41 | Morocco | | 2003 | Lower-middle income | 6180 |
|  | | **South Asia** | | | |
| 42 | Bangladesh | | 2011 | Low income | 8753 |
| 43 | India | | 2005 | Lower-middle income | 51555 |
| 44 | Maldives | | 2009 | Lower-middle income | 3817 |
| 45 | Nepal | | 2011 | Low income | 5306 |
| 46 | Pakistan | | 2012 | Lower-middle income | 11763 |

+ Listing obtained from <http://www.preventionweb.net/english/hyogo/gar/2011/en/bgdocs/Annexes/GAR%2011%20Annex%201%20World%20Bank%20country%20classification.pdf> (Last accessed on 3^rd^ February 2017)
