# Supplementary material for: Socioeconomic inequalities in skilled birth attendance and child stunting in selected low and middle income countries: Wealth quintiles or deciles?
Source: PLoS One. 2017 May 3;12(5):e0174823. doi: 10.1371/journal.pone.0174823 (PMC5414946; doi:10.1371/journal.pone.0174823)
Supplement: S2 Table — (DOCX) [file pone.0174823.s002.docx]

**S2 Table. Prevalence of stunting among children under five years of age, by wealth quintiles and deciles.**

| **Country** | **Year** | **National coverage** | **Deciles** | | | | | | | | | |  | **Quintiles** | | | | |
| --- | --- | --- | --- | --- | --- | --- | --- | --- | --- | --- | --- | --- | --- | --- | --- | --- | --- | --- |
|  |  |  | **D1** | **D2** | **D3** | **D4** | **D5** | **D6** | **D7** | **D8** | **D9** | **D10** |  | **Q1** | **Q2** | **Q3** | **Q4** | **Q5** |
| Bangladesh | 2011 | 41.2 | 55.8 | 50.6 | 47.0 | 44.8 | 41.3 | 38.9 | 36.1 | 36.1 | 29.8 | 21.3 |  | 53.5 | 45.9 | 40.1 | 36.1 | 25.7 |
| Benin | 2011 | 43.7 | 49.1 | 48.4 | 46.8 | 46.0 | 46.6 | 42.0 | 42.5 | 42.0 | 38.8 | 34.6 |  | 48.8 | 46.4 | 44.4 | 42.3 | 36.9 |
| Bolivia | 2008 | 27.1 | 48.3 | 42.2 | 38.4 | 30.6 | 22.8 | 21.0 | 15.0 | 13.6 | 6.0 | 6.6 |  | 45.5 | 34.6 | 21.9 | 14.3 | 6.2 |
| Burkina Faso | 2010 | 34.5 | 42.4 | 41.5 | 37.2 | 36.5 | 40.1 | 35.0 | 34.9 | 31.4 | 21.6 | 12.6 |  | 41.9 | 36.8 | 37.5 | 33.2 | 17.9 |
| Burundi | 2010 | 57.9 | 68.9 | 70.3 | 61.5 | 57.0 | 64.4 | 57.4 | 57.3 | 55.9 | 50.6 | 30.1 |  | 69.6 | 59.3 | 60.7 | 56.6 | 41.5 |
| Cambodia | 2010 | 39.1 | 51.3 | 47.4 | 43.4 | 44.8 | 41.8 | 36.1 | 35.9 | 31.9 | 25.7 | 17.8 |  | 49.4 | 44.1 | 38.7 | 34.0 | 21.9 |
| Cameroon | 2011 | 32.0 | 50.0 | 47.4 | 42.0 | 39.6 | 30.7 | 28.3 | 23.0 | 20.7 | 16.0 | 7.2 |  | 48.7 | 40.8 | 29.6 | 21.9 | 12.2 |
| Chad | 2004 | 44.6 | 54.6 | 51.2 | 46.5 | 43.1 | 52.1 | 44.2 | 48.8 | 36.0 | 40.2 | 29.3 |  | 52.8 | 44.7 | 47.7 | 42.4 | 34.9 |
| Colombia | 2010 | 13.1 | 23.1 | 15.3 | 13.0 | 13.2 | 12.4 | 11.3 | 9.0 | 10.3 | 6.2 | 7.0 |  | 19.5 | 13.1 | 11.9 | 9.6 | 6.6 |
| Comoros | 2012 | 29.6 | 43.1 | 31.5 | 34.9 | 29.4 | 22.8 | 27.2 | 24.5 | 28.4 | 25.1 | 18.0 |  | 37.3 | 32.2 | 25.3 | 26.5 | 22.1 |
| Congo Brazz | 2011 | 23.1 | 34.8 | 31.1 | 24.6 | 28.4 | 29.6 | 21.8 | 16.8 | 13.5 | 14.6 | 1.6 |  | 33.0 | 26.7 | 25.6 | 15.3 | 8.7 |
| Congo DR | 2007 | 44.5 | 43.0 | 48.0 | 46.6 | 46.8 | 59.1 | 46.9 | 52.8 | 42.5 | 29.6 | 18.1 |  | 45.3 | 46.7 | 53.8 | 47.5 | 24.5 |
| Cote dIvoire | 2011 | 29.8 | 42.5 | 36.3 | 41.8 | 28.9 | 25.5 | 28.2 | 26.8 | 23.4 | 15.2 | 13.2 |  | 39.2 | 35.4 | 26.8 | 25.1 | 14.4 |
| Dominican Rep | 2007 | 9.8 | 20.1 | 13.1 | 11.6 | 7.8 | 8.0 | 6.8 | 7.6 | 7.5 | 4.2 | 4.7 |  | 16.4 | 9.7 | 7.4 | 7.6 | 4.4 |
| Egypt | 2008 | 28.9 | 31.8 | 27.3 | 32.8 | 28.4 | 27.4 | 27.0 | 32.4 | 27.3 | 28.5 | 25.1 |  | 29.5 | 30.6 | 27.2 | 29.9 | 27.0 |
| Ethiopia | 2011 | 44.3 | 46.7 | 50.9 | 48.0 | 47.3 | 46.0 | 46.0 | 45.5 | 45.2 | 34.2 | 21.1 |  | 48.8 | 47.6 | 46.0 | 45.3 | 29.1 |
| Gabon | 2012 | 16.0 | 35.4 | 24.9 | 16.4 | 19.8 | 14.5 | 7.7 | 13.5 | 11.6 | 7.4 | 3.4 |  | 29.6 | 18.1 | 11.3 | 12.6 | 5.4 |
| Guinea | 2012 | 30.9 | 36.9 | 30.1 | 43.1 | 36.2 | 34.8 | 33.3 | 28.0 | 21.5 | 20.7 | 9.7 |  | 33.4 | 39.7 | 34.0 | 25.1 | 15.1 |
| Haiti | 2012 | 20.9 | 32.1 | 28.8 | 26.5 | 24.0 | 18.2 | 21.6 | 15.6 | 14.7 | 6.9 | 5.6 |  | 30.4 | 25.3 | 19.9 | 15.2 | 6.4 |
| Honduras | 2011 | 22.3 | 46.9 | 36.6 | 30.0 | 20.5 | 16.2 | 14.8 | 11.7 | 11.4 | 9.7 | 4.3 |  | 42.2 | 25.3 | 15.5 | 11.5 | 7.5 |
| India | 2005 | 48.0 | 60.8 | 58.9 | 55.1 | 53.7 | 50.8 | 46.7 | 42.9 | 38.4 | 29.8 | 20.5 |  | 59.9 | 54.4 | 48.8 | 40.8 | 25.6 |
| Jordan | 2012 | 7.6 | 14.8 | 13.2 | 8.4 | 5.4 | 5.6 | 6.9 | 7.5 | 5.8 | 2.6 | 0.1 |  | 13.9 | 6.9 | 6.2 | 6.7 | 1.8 |
| Kenya | 2008 | 35.3 | 44.8 | 43.6 | 42.8 | 35.2 | 38.4 | 29.5 | 30.5 | 28.0 | 31.0 | 18.5 |  | 44.2 | 39.3 | 34.2 | 29.2 | 25.2 |
| Kyrgyzstan | 2012 | 17.8 | 16.4 | 19.3 | 19.5 | 18.2 | 18.6 | 15.6 | 17.8 | 15.6 | 17.3 | 20.0 |  | 17.9 | 18.8 | 17.0 | 16.8 | 18.7 |
| Lesotho | 2009 | 37.6 | 47.4 | 42.3 | 46.3 | 38.0 | 38.8 | 40.2 | 26.8 | 29.7 | 28.8 | 27.4 |  | 44.8 | 42.4 | 39.4 | 28.1 | 28.1 |
| Liberia | 2013 | 30.2 | 33.8 | 32.5 | 31.9 | 34.3 | 32.6 | 36.7 | 23.8 | 28.1 | 24.0 | 12.3 |  | 33.2 | 33.0 | 34.8 | 25.9 | 19.3 |
| Madagascar | 2008 | 50.4 | 50.1 | 46.0 | 52.3 | 55.6 | 50.5 | 55.2 | 53.5 | 50.7 | 49.5 | 35.2 |  | 48.0 | 53.9 | 52.7 | 52.2 | 43.4 |
| Malawi | 2010 | 47.1 | 60.8 | 51.8 | 51.1 | 51.0 | 48.1 | 44.7 | 46.1 | 47.5 | 37.2 | 32.4 |  | 55.8 | 51.1 | 46.5 | 46.8 | 35.1 |
| Maldives | 2009 | 18.0 | 21.2 | 21.3 | 21.7 | 22.3 | 18.3 | 14.3 | 16.4 | 15.1 | 17.0 | 12.3 |  | 21.3 | 22.0 | 16.4 | 15.7 | 14.3 |
| Morocco | 2003 | 22.5 | 35.7 | 34.8 | 28.2 | 20.5 | 23.3 | 16.8 | 15.6 | 14.5 | 15.2 | 10.1 |  | 35.3 | 24.5 | 19.8 | 15.0 | 12.6 |
| Mozambique | 2011 | 42.8 | 53.3 | 48.2 | 46.9 | 48.9 | 47.8 | 45.4 | 36.7 | 38.4 | 30.2 | 16.5 |  | 50.8 | 47.9 | 46.5 | 37.6 | 24.2 |
| Namibia | 2006 | 28.7 | 40.9 | 32.9 | 38.1 | 29.4 | 27.8 | 31.9 | 27.5 | 23.6 | 18.7 | 3.6 |  | 37.5 | 34.0 | 29.6 | 25.7 | 12.1 |
| Nepal | 2011 | 40.3 | 56.6 | 55.6 | 46.0 | 44.4 | 41.3 | 28.2 | 35.5 | 24.9 | 30.5 | 14.9 |  | 56.2 | 45.3 | 35.0 | 30.0 | 24.9 |
| Niger | 2012 | 43.3 | 44.4 | 47.1 | 47.8 | 49.3 | 39.8 | 42.6 | 48.4 | 41.9 | 41.7 | 25.2 |  | 45.9 | 48.5 | 41.2 | 45.5 | 34.2 |
| Nigeria | 2013 | 36.7 | 53.5 | 54.2 | 47.1 | 44.1 | 36.8 | 33.1 | 27.2 | 25.0 | 22.1 | 13.0 |  | 53.9 | 45.7 | 35.0 | 26.1 | 18.0 |
| Pakistan | 2012 | 44.4 | 57.9 | 66.2 | 53.7 | 57.2 | 42.3 | 37.9 | 41.1 | 32.6 | 27.4 | 18.2 |  | 61.9 | 55.6 | 40.1 | 37.7 | 23.1 |
| Rwanda | 2010 | 44.0 | 53.1 | 54.2 | 53.9 | 48.7 | 47.0 | 43.4 | 42.1 | 35.2 | 31.8 | 19.2 |  | 53.6 | 51.2 | 45.1 | 38.7 | 25.8 |
| Senegal | 2012 | 18.4 | 27.3 | 23.6 | 24.5 | 21.4 | 17.2 | 16.0 | 13.8 | 11.0 | 8.3 | 13.4 |  | 25.4 | 23.0 | 16.6 | 12.6 | 10.6 |
| Sierra Leone | 2008 | 35.5 | 32.9 | 36.2 | 46.0 | 43.3 | 33.0 | 38.9 | 36.9 | 33.9 | 23.8 | 19.2 |  | 34.6 | 44.5 | 35.9 | 35.4 | 21.8 |
| Tajikistan | 2012 | 26.1 | 32.1 | 32.4 | 30.2 | 28.5 | 22.1 | 23.6 | 26.9 | 22.6 | 21.2 | 20.0 |  | 32.3 | 29.3 | 22.8 | 24.8 | 20.6 |
| Tanzania | 2010 | 41.6 | 50.5 | 44.2 | 43.1 | 46.2 | 45.5 | 42.2 | 42.6 | 35.9 | 31.4 | 22.4 |  | 47.9 | 44.6 | 43.8 | 39.3 | 27.3 |
| Timor-Leste | 2009 | 57.6 | 63.5 | 61.5 | 64.2 | 64.7 | 57.5 | 60.8 | 57.2 | 52.8 | 51.8 | 40.5 |  | 62.6 | 64.4 | 59.2 | 55.1 | 46.5 |
| Turkey | 2003 | 15.2 | 34.4 | 26.2 | 17.0 | 15.6 | 13.4 | 10.4 | 7.1 | 4.1 | 4.0 | 4.6 |  | 30.7 | 16.4 | 12.0 | 5.6 | 4.2 |
| Uganda | 2011 | 33.2 | 39.4 | 32.4 | 31.0 | 29.6 | 50.0 | 41.1 | 38.5 | 22.1 | 25.7 | 17.0 |  | 35.7 | 30.3 | 45.7 | 30.4 | 21.6 |
| Zambia | 2007 | 45.3 | 47.9 | 48.3 | 48.8 | 52.4 | 47.9 | 46.5 | 43.4 | 39.4 | 41.4 | 26.3 |  | 48.1 | 50.7 | 47.2 | 41.4 | 33.9 |
| Zimbabwe | 2010 | 31.1 | 35.1 | 35.4 | 33.4 | 27.5 | 35.7 | 32.5 | 28.1 | 29.0 | 23.4 | 24.3 |  | 35.3 | 30.7 | 34.1 | 28.5 | 23.7 |
