# Supplementary material for: Socioeconomic inequalities in skilled birth attendance and child stunting in selected low and middle income countries: Wealth quintiles or deciles?
Source: PLoS One. 2017 May 3;12(5):e0174823. doi: 10.1371/journal.pone.0174823 (PMC5414946; doi:10.1371/journal.pone.0174823)
Supplement: S3 Table — (DOCX) [file pone.0174823.s003.docx]

**S3 Table. Coverage with skilled birth attendants, by wealth quintiles and deciles.**

| **Country** | **Year** | **National coverage** | **Deciles** | | | | | | | | | |  | **Quintiles** | | | | |
| --- | --- | --- | --- | --- | --- | --- | --- | --- | --- | --- | --- | --- | --- | --- | --- | --- | --- | --- |
|  |  |  | **D1** | **D2** | **D3** | **D4** | **D5** | **D6** | **D7** | **D8** | **D9** | **D10** |  | **Q1** | **Q2** | **Q3** | **Q4** | **Q5** |
| Bangladesh | 2011 | 27.7 | 8.2 | 11.0 | 12.7 | 17.7 | 20.0 | 27.8 | 32.5 | 42.0 | 49.3 | 73.7 |  | 9.4 | 15.1 | 23.8 | 37.3 | 61.1 |
| Benin | 2011 | 84.1 | 60.3 | 68.6 | 75.5 | 81.4 | 83.2 | 86.6 | 95.0 | 98.1 | 98.6 | 99.0 |  | 64.4 | 78.5 | 84.9 | 96.6 | 98.8 |
| Bolivia | 2008 | 71.1 | 31.5 | 45.9 | 57.3 | 75.3 | 77.0 | 86.0 | 89.0 | 95.5 | 98.9 | 98.4 |  | 37.9 | 65.9 | 81.4 | 92.0 | 98.7 |
| Burkina Faso | 2010 | 67.1 | 45.4 | 47.7 | 55.2 | 59.6 | 65.3 | 71.5 | 75.6 | 80.0 | 90.4 | 97.3 |  | 46.6 | 57.4 | 68.4 | 77.8 | 93.3 |
| Burundi | 2010 | 60.3 | 49.4 | 52.0 | 52.1 | 55.1 | 58.3 | 57.0 | 57.7 | 64.8 | 71.3 | 92.7 |  | 50.7 | 53.6 | 57.6 | 61.4 | 80.8 |
| Cambodia | 2010 | 71.0 | 44.4 | 53.3 | 60.4 | 67.1 | 71.4 | 77.7 | 82.0 | 91.5 | 94.2 | 99.6 |  | 48.7 | 63.6 | 74.6 | 86.5 | 96.7 |
| Cameroon | 2011 | 63.6 | 14.6 | 23.2 | 48.8 | 60.2 | 72.9 | 78.2 | 88.0 | 91.2 | 97.1 | 96.5 |  | 19.1 | 54.4 | 75.5 | 89.5 | 96.8 |
| Chad | 2004 | 20.7 | 1.0 | 8.2 | 8.8 | 15.3 | 9.5 | 17.0 | 14.7 | 25.7 | 40.8 | 69.8 |  | 4.9 | 12.3 | 13.6 | 20.1 | 54.5 |
| Colombia | 2010 | 94.6 | 76.9 | 91.9 | 94.9 | 97.9 | 98.5 | 98.9 | 99.0 | 99.6 | 99.2 | 99.6 |  | 83.7 | 96.4 | 98.7 | 99.3 | 99.4 |
| Comoros | 2012 | 82.2 | 60.0 | 72.8 | 74.7 | 83.1 | 87.1 | 85.7 | 94.2 | 93.1 | 89.4 | 96.7 |  | 66.4 | 78.8 | 86.3 | 93.7 | 92.7 |
| Congo Brazz | 2011 | 93.6 | 74.9 | 87.4 | 92.4 | 97.2 | 97.8 | 97.3 | 97.9 | 98.7 | 99.9 | 99.0 |  | 80.9 | 95.0 | 97.5 | 98.3 | 99.5 |
| Congo DR | 2007 | 74.0 | 57.6 | 62.4 | 63.3 | 61.2 | 73.8 | 72.5 | 79.1 | 91.6 | 97.2 | 98.4 |  | 59.9 | 62.2 | 73.2 | 85.1 | 97.7 |
| Cote dIvoire | 2011 | 59.4 | 28.2 | 42.2 | 45.5 | 57.6 | 50.1 | 58.9 | 74.7 | 83.9 | 87.1 | 96.3 |  | 35.0 | 51.8 | 54.5 | 79.0 | 91.2 |
| Dominican Rep | 2007 | 97.8 | 91.9 | 97.8 | 98.3 | 99.0 | 98.6 | 98.4 | 98.6 | 99.7 | 99.5 | 98.7 |  | 95.0 | 98.6 | 98.5 | 99.1 | 99.1 |
| Egypt | 2008 | 78.9 | 52.4 | 58.0 | 66.5 | 73.8 | 79.6 | 85.9 | 89.6 | 91.9 | 96.0 | 98.2 |  | 55.2 | 70.1 | 82.8 | 90.7 | 96.9 |
| Ethiopia | 2011 | 10.8 | 2.5 | 1.8 | 4.6 | 3.0 | 4.1 | 4.1 | 5.6 | 11.4 | 26.9 | 76.1 |  | 2.1 | 3.8 | 4.1 | 8.5 | 46.3 |
| Gabon | 2012 | 90.0 | 62.5 | 86.4 | 88.7 | 95.0 | 93.2 | 94.6 | 94.3 | 96.2 | 94.8 | 95.6 |  | 75.6 | 91.8 | 93.8 | 95.2 | 95.2 |
| Guinea | 2012 | 41.1 | 9.3 | 14.2 | 21.2 | 29.1 | 31.9 | 39.3 | 47.2 | 76.7 | 88.1 | 94.5 |  | 11.7 | 24.9 | 35.6 | 60.9 | 91.0 |
| Haiti | 2012 | 37.3 | 9.0 | 10.2 | 15.7 | 25.7 | 32.9 | 44.0 | 48.6 | 61.2 | 73.3 | 84.8 |  | 9.6 | 20.5 | 38.4 | 54.5 | 78.1 |
| Honduras | 2011 | 82.9 | 50.1 | 66.9 | 75.8 | 86.1 | 89.4 | 92.7 | 95.0 | 97.4 | 98.0 | 98.9 |  | 57.7 | 80.8 | 91.1 | 96.1 | 98.4 |
| India | 2005 | 46.6 | 16.9 | 22.1 | 28.8 | 34.9 | 45.7 | 52.6 | 61.5 | 73.6 | 84.2 | 94.3 |  | 19.4 | 31.8 | 49.0 | 67.2 | 88.8 |
| Jordan | 2012 | 99.6 | 98.7 | 99.1 | 100.0 | 100.0 | 99.9 | 100.0 | 100.0 | 99.0 | 100.0 | 100.0 |  | 98.9 | 100.0 | 100.0 | 99.5 | 100.0 |
| Kenya | 2008 | 43.8 | 14.5 | 26.5 | 26.2 | 36.6 | 35.4 | 50.2 | 51.2 | 54.4 | 75.4 | 87.8 |  | 20.3 | 31.1 | 42.3 | 52.9 | 81.4 |
| Kyrgyzstan | 2012 | 99.1 | 99.4 | 98.9 | 99.5 | 99.6 | 98.1 | 99.5 | 97.7 | 99.5 | 99.8 | 99.7 |  | 99.2 | 99.6 | 98.8 | 98.5 | 99.8 |
| Lesotho | 2009 | 61.5 | 30.8 | 38.9 | 43.7 | 56.6 | 54.7 | 67.1 | 75.2 | 81.9 | 88.4 | 91.1 |  | 34.6 | 50.1 | 60.9 | 78.5 | 89.6 |
| Liberia | 2013 | 61.1 | 40.3 | 46.5 | 45.9 | 55.2 | 60.9 | 63.8 | 69.2 | 82.5 | 85.8 | 93.1 |  | 43.2 | 50.4 | 62.3 | 75.8 | 89.0 |
| Madagascar | 2008 | 43.9 | 20.3 | 23.6 | 25.3 | 31.6 | 38.0 | 48.1 | 52.8 | 68.1 | 86.8 | 94.7 |  | 21.9 | 28.4 | 42.8 | 60.1 | 90.1 |
| Malawi | 2010 | 71.3 | 62.3 | 64.3 | 66.5 | 64.4 | 66.1 | 69.1 | 77.6 | 76.0 | 86.0 | 91.8 |  | 63.3 | 65.5 | 67.6 | 76.8 | 88.5 |
| Maldives | 2009 | 94.8 | 85.9 | 91.2 | 91.2 | 94.0 | 95.4 | 95.3 | 98.3 | 98.8 | 100.0 | 98.7 |  | 88.7 | 92.6 | 95.3 | 98.5 | 99.3 |
| Morocco | 2003 | 62.6 | 23.2 | 36.4 | 42.5 | 57.1 | 63.1 | 77.0 | 83.3 | 88.8 | 93.9 | 97.0 |  | 29.4 | 49.4 | 70.3 | 86.0 | 95.4 |
| Mozambique | 2011 | 54.3 | 32.2 | 30.7 | 33.8 | 41.7 | 49.2 | 56.0 | 70.4 | 77.9 | 87.6 | 91.9 |  | 31.5 | 37.7 | 52.7 | 74.2 | 89.5 |
| Namibia | 2006 | 81.4 | 56.0 | 64.9 | 73.8 | 72.8 | 84.8 | 86.2 | 92.2 | 95.3 | 96.4 | 99.3 |  | 59.9 | 73.3 | 85.5 | 93.7 | 97.7 |
| Nepal | 2011 | 36.0 | 7.9 | 14.1 | 20.7 | 27.0 | 32.8 | 39.4 | 48.5 | 57.1 | 75.8 | 90.4 |  | 10.7 | 23.7 | 36.0 | 53.0 | 81.5 |
| Niger | 2012 | 29.3 | 10.0 | 13.5 | 17.5 | 19.1 | 18.8 | 24.0 | 24.9 | 34.0 | 56.5 | 87.9 |  | 11.8 | 18.3 | 21.4 | 29.2 | 71.0 |
| Nigeria | 2013 | 38.1 | 3.8 | 7.6 | 12.2 | 23.0 | 34.2 | 45.8 | 55.8 | 68.4 | 81.2 | 90.1 |  | 5.7 | 17.3 | 39.9 | 62.0 | 85.3 |
| Pakistan | 2012 | 52.1 | 29.4 | 30.2 | 35.8 | 40.2 | 48.4 | 53.9 | 61.4 | 77.8 | 78.9 | 92.1 |  | 29.8 | 38.1 | 51.2 | 68.9 | 85.2 |
| Rwanda | 2010 | 69.0 | 58.8 | 63.8 | 61.2 | 65.8 | 66.9 | 66.5 | 72.7 | 72.5 | 78.7 | 93.7 |  | 61.2 | 63.5 | 66.7 | 72.6 | 85.9 |
| Senegal | 2012 | 75.7 | 33.7 | 49.1 | 68.4 | 76.5 | 82.5 | 90.1 | 93.8 | 92.2 | 96.7 | 98.7 |  | 41.5 | 72.4 | 86.2 | 93.1 | 97.6 |
| Sierra Leone | 2008 | 42.4 | 24.8 | 31.5 | 33.7 | 37.1 | 37.9 | 39.5 | 43.4 | 55.3 | 67.2 | 76.0 |  | 28.1 | 35.4 | 38.7 | 49.0 | 71.4 |
| Tajikistan | 2012 | 87.4 | 68.6 | 79.5 | 84.5 | 85.6 | 87.6 | 91.5 | 94.4 | 92.8 | 96.9 | 95.1 |  | 73.9 | 85.1 | 89.5 | 93.6 | 96.1 |
| Tanzania | 2010 | 50.6 | 31.3 | 35.6 | 34.8 | 37.1 | 43.5 | 47.8 | 55.8 | 65.5 | 86.5 | 94.9 |  | 33.1 | 35.9 | 45.7 | 60.7 | 90.3 |
| Timor-Leste | 2009 | 29.9 | 9.2 | 12.0 | 14.1 | 14.2 | 19.1 | 23.9 | 29.2 | 47.7 | 59.2 | 79.7 |  | 10.6 | 14.1 | 21.6 | 38.4 | 69.0 |
| Turkey | 2003 | 83.0 | 55.0 | 62.9 | 77.4 | 85.0 | 93.0 | 90.5 | 94.1 | 98.0 | 99.2 | 99.2 |  | 58.5 | 80.8 | 91.8 | 96.0 | 99.2 |
| Uganda | 2011 | 58.0 | 40.2 | 46.9 | 48.6 | 49.9 | 51.7 | 56.4 | 52.8 | 67.3 | 84.1 | 92.9 |  | 43.5 | 49.2 | 54.1 | 59.6 | 88.4 |
| Zambia | 2007 | 46.5 | 26.1 | 27.7 | 25.2 | 30.1 | 33.5 | 39.5 | 60.3 | 82.6 | 88.6 | 94.0 |  | 26.8 | 27.7 | 36.5 | 71.4 | 91.3 |
| Zimbabwe | 2010 | 66.2 | 47.5 | 47.6 | 53.4 | 58.6 | 58.7 | 68.6 | 74.3 | 87.3 | 90.1 | 91.4 |  | 47.5 | 55.9 | 63.6 | 80.9 | 90.6 |
