# Supplementary material for: Socioeconomic inequalities in skilled birth attendance and child stunting in selected low and middle income countries: Wealth quintiles or deciles?
Source: PLoS One. 2017 May 3;12(5):e0174823. doi: 10.1371/journal.pone.0174823 (PMC5414946; doi:10.1371/journal.pone.0174823)
Supplement: S1 Supporting Information — (DOCX) [file pone.0174823.s004.docx]

**S1 Supporting Information. Sample size calculations.**

**Technical Notes**

- Margins of error refer to the error above or below the coverage estimate; for example, 50% coverage with a margin of 4 pct points indicates that coverage lies between 46% and 54%.
- Coverage values refer to expected coverage for a particular quintile or decile, not to coverage in the national sample.
- The tables are labeled in terms of coverage, but results are valid for other outcomes (e.g. prevalence).
- The total sample sizes (column B) refer to the number of mothers or children who make up the denominator in the national survey. For example, for ORS the denominator is children with diarrhea.
- Users may have to allow for design effect due to clustered samples; these are not included in the present calculations.
- Users may have to allow for the fact that there tend to be more children in the poorest than in the richest quintile or decile (see below).

**Formula**

For 95% confidence intervals: Precision refers to the margin of error in percent points to each side of the estimate. For example, a precision of 4 pct points around a 50% coverage estimate means that the true value will be between 46% and 54% (95% of the time).

Precision = square root (3.84 * coverage * (1-coverage)/sample size in each group)

Note: 3.84 is the parameter corresponding to a 95% confidence interval

Note: a sample size of 500 children, for example, is estimated to include 50 children in each decile and 100 in each quintile (This ignores the possibility that there may be more children in the poorer groups because of higher fertility)

**Calculations**

(double click on object to access)
